# Experimental and Analytical Tools for the Chemical Recycling of Engineering Plastics – A Multi‐Scale Pyrolysis Study on Polydicyclopentadiene

**DOI:** 10.1002/marc.202500161

**Published:** 2025-05-29

**Authors:** Michael Zeller, Salar Tavakkol, Dieter Stapf

**Affiliations:** ^1^ Institute for Technical Chemistry Karlsruhe Institute of Technology Kaiserstrasse 12 76131 Karlsruhe Germany

**Keywords:** chemical recycling, circular economy, gas chromatography, polydicyclopentadiene, pyrolysis, thermogravimetry

## Abstract

The creation of a circular economy for plastics is essential for a sustainable future. Currently, established recycling processes are not universally applicable. Pyrolysis can complement current recycling through the conversion of complex plastic wastes to condensates and gases for reintroduction into chemical industry processes. A multi‐scale approach for the characterization of pyrolysis properties is presented using the example of polydicyclopentadiene. Pyrolysis‐GC‐MS (µg‐scale), thermogravimetric (mg‐scale), and lab‐scale (g‐scale) pyrolysis investigations are complemented by high‐resolution product analytics. In Py‐GC‐MS, 1,3‐cyclopentadiene is abundant, hinting at depolymerization as a dominant decomposition mechanism. In thermogravimetry, approx. 20 mass‐% of the sample is converted to solids. On lab‐scale, secondary reactions influence the product yield and spectrum significantly, indicated by a solid yield of ≈40 mass‐%. Polydicyclopentadiene exhibits a broad product spectrum comprising unsaturated hydrocarbons and aromatics. In the condensed product, no 1,3‐cyclopentadiene is detected, indicating recombination or derivatization reactions. Significant solid residue formation emphasizes scaling effects. The employed analytics provide comprehensive analyses of condensed and non‐condensed products. With the presented approach, primary and secondary mechanisms can be resolved and crucial process parameters identified. In this way, a powerful toolset for the development of robust pyrolysis processes that aid in achieving circularity for plastics is provided.

## Graphical abstract

The presented work demonstrates the importance of a comprehensive assessment of engineering plastics pyrolysis on the example of Polydicyclopentadiene. Analyses in µg‐, mg‐, and lab‐scale provide insight into mechanisms, yields, and products. An analytical and experimental toolset is showcased, by which the foundation for the assessment of pyrolysis as a circular technology is laid.

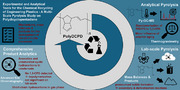

## 1 Introduction

To achieve the sustainability goals set by the United Nations and to establish safe operating spaces within the boundaries of the planet, sustainable solutions for plastic waste are needed. This comprises the full life cycle of these materials. The use of sustainable feedstocks for polymer production, extending polymer lifetime, and high‐value recycling are essential.^[^ ^1^ ^]^ For many mass plastics such as Polyethylene, Polypropylene, Polystyrene, and Polyethylene terephthalate, mechanical and chemical recycling processes have already been established at a large scale. For engineering plastics, however, such processes are scarce. The manufacturing of engineering plastics includes the use of special monomers and polymerization procedures, as well as blending with additives to achieve the desired specialized properties. This is detrimental to the recycling of these materials. Mechanical recycling based on sorting and re‐melting is either unsuitable for thermosets or significantly hindered by the presence of additives and other contaminants in plastic waste. Solvent‐based recycling approaches struggle with similar hindrances. Novel approaches that aim at the potential regeneration of monomers and oligomers for reprocessing have emerged only very recently.^[^ ^2^, ^3^, ^4^ ^]^ A potential option to recover valuable products from complex plastic wastes is thermo‐chemical recycling. In processes like gasification or pyrolysis, polymers are broken down into significantly smaller molecules. Additives and contaminants can be discharged from the product streams. The products of thermo‐chemical recycling can then be reintegrated into process chains of the chemical industry, replacing fossil feedstocks and supporting the transition to a circular economy. While gasification converts plastic waste into a synthesis gas comprised of H_2_, CO, CO_2_, and H_2_O in sub‐stoichiometric combustion conditions, pyrolysis decomposes plastic waste into solid, condensable, and gaseous products in an O‐free atmosphere. These products could be fed into downstream processes such as steam crackers, fluid catalytic crackers, and gasifiers, among other options. However, pyrolysis products must first fulfill strict product specifications that include limits for, e.g., O, N, halogen, or aromatics content.^[^ ^5^ ^]^

The thermo‐chemical decomposition behavior is highly dependent on the employed feedstock. Therefore, the recycling potential of a certain plastic type must be evaluated individually. This applies in particular to engineering plastics. Elucidation of the decomposition characteristics requires detailed analyses of the condensed and non‐condensed product phases. For such analyses, gas chromatography (GC) is a powerful tool. The high number of analytes in each product phase requires the employment of specialized GC setups, detectors, and methods.

In the presented work, we demonstrate the value of multi‐scale experimental setups and comprehensive analytics for the assessment of complex plastic pyrolysis products and processes. The pyrolysis properties and the circularity potential of Polydicyclopentadiene (PDCPD) are investigated. PDCPD is used for large plastic parts with high durability and mechanical requirements, e.g. in trucks and heavy machinery. The polymer is synthesized from Dicyclopentadiene (DCPD) by ring‐opening metathesis polymerization and exhibits thermoset properties. Mechanical recycling is thus inherently linked to downcycling. The polymeric backbone of PDCPD contains no heteroatoms. This is highly advantageous for chemical recycling since no heteroatoms must be removed for further downstream processing. Potentially, PDCPD pyrolysis products could be integrated into recycling routes similar to those already established for polyolefins due to their expected similarity in composition.

As mentioned, pyrolysis yields solid, condensable, and gaseous products. The distribution of these product phases and the chemical composition of each depend on the pyrolyzed polymer and the process conditions. For PDCPD, literature information on the pyrolytic decomposition characteristics is scarce. Mühlebach et al. have found that PDCPD pyrolysis yields 70% DCPD besides other cyclic alkanes and aromatics, hinting at depolymerization as the predominant mechanism.^[^ ^6^ ^]^ In the context of recycling, this is promising. With controlled depolymerization, closed‐loop recycling of PDCPD is possible. In the presented work, we investigated the pyrolysis of PDCPD by Pyrolysis‐Gas‐Chromatography‐Mass‐Spectroscopy (Py‐GC‐MS), thermogravimetric analysis (TGA), and lab‐scale pyrolysis. Advanced gas chromatographic analyses resolve the predominant compounds in the gaseous and condensed phases. The primary decomposition mechanisms, the influence of secondary reactions, and the heat‐ and mass‐transfer influence are evaluated. We present mass balances, product spectra, and compound analyses for experiments from µg to g‐scale. This allows for the assessment of pyrolysis as a recycling approach for PDCPD.

## 2 Results and Discussion

### 2.1 Thermogravimetry

**Figure 1:**
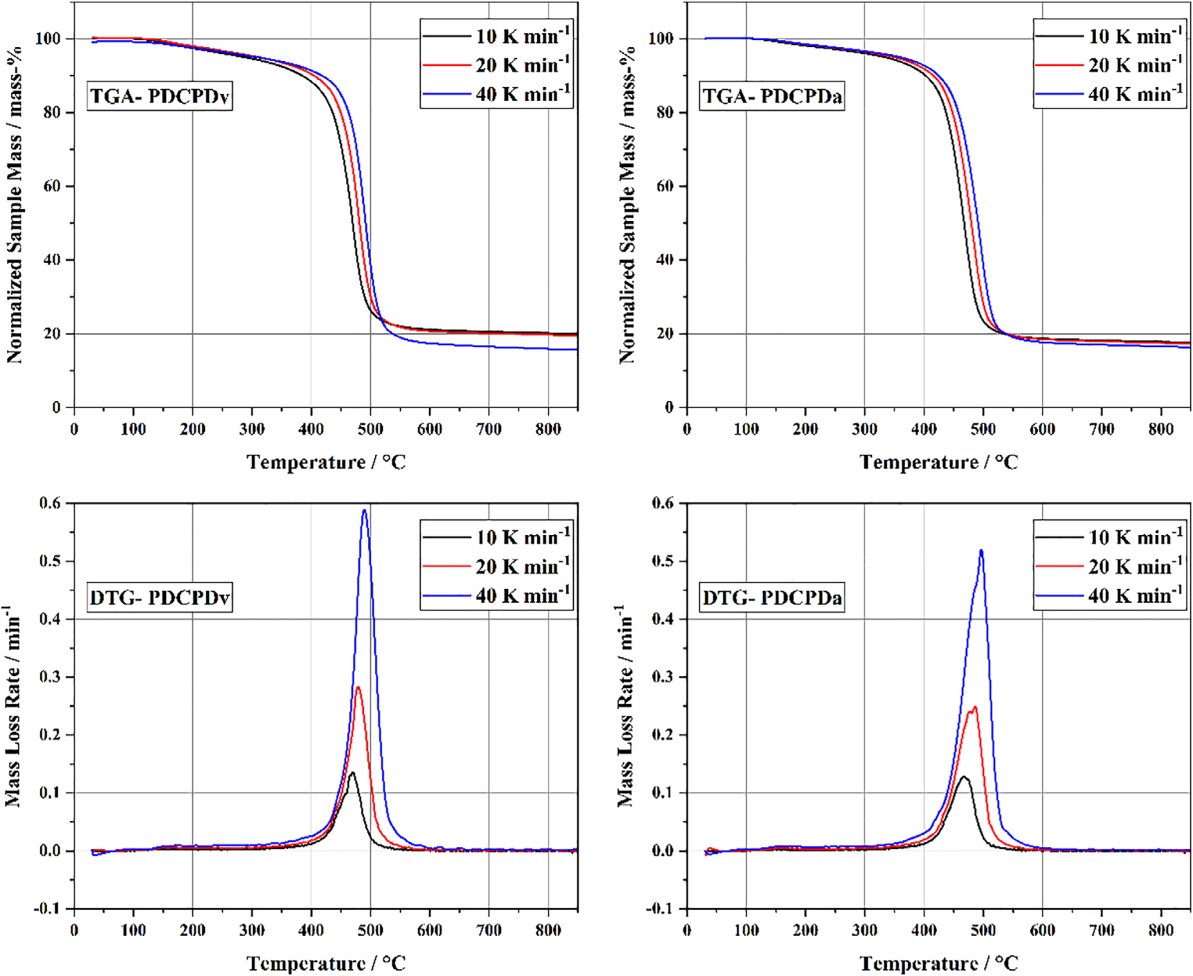
Thermogravimetric mass loss curves (TGA) and derivatives (DTG) of PDCPDv and PDCPDa.

Non‐pretreated (PDCPDv) and artificially aged (PDCPDa) samples were investigated in TGA. The thermogravimetric mass loss profiles and their derivatives are displayed in **Figure** **1**.

The TGA of both samples shows volatile formation in two stages. The general degradation behavior appears independent of the heating rate and the sample aging history. The first volatile formation stage starts around 100 °C. During the first stage, ≈10% of the sample mass is volatilized. The mass loss occurs comparatively slowly. This is particularly evident from the DTG curves, which show low intensity. This initial mass loss could be due to the volatilization of adsorbed gases, additives, or unreacted monomers. It is also possible that slight polymer degradation occurs within this temperature range. However, since the exact composition of the polymer, including the content and types of catalysts and additives, is unknown to the authors, the cause and precise nature of the first‐stage mass loss remain unclear. The second volatilization stage lies between ≈350 and 550 °C. This stage exhibits intense mass loss, amounting to ≈70% of the sample mass. Above 550 °C, no significant additional mass loss is observed. For PDCPDv, the amount of solid residue is 19.9, 19.5, and 15.4 mass‐% for the heating rates 10, 20, and 40 K min^−1^, respectively. For PDCPDa, the amount of solid residue is 17.5, 17.3, and 16.3 mass‐% for the heating rates 10, 20, and 40 K min^−1^, respectively. The heating rate and the sample age appear to influence the residue formation slightly. Faster heating of the sample results in lower residue formation. This may be due to a faster progression of the decomposition and faster evaporation of products from the crucible. Once evaporated from the crucible, the decomposition products are no longer available for secondary reactions such as the formation of residue.

With an increased heating rate, a slight shift of the mass loss curves to higher temperatures is observable. This is attributed to the heat transfer characteristics of the TGA system and is thus classified as a minor artifact.

Besides the residue formation, the observed differences between the virgin and the aged PDCPD samples are marginal. It is known that PDCPD is prone to oxidation.^[^ ^7^, ^8^ ^]^ Vidavsky et al. and Mühlebach et al. have shown that the glass transition temperature of PDCPD increases when aged and exposed to thermal stress.^[^ ^6^, ^9^ ^]^ Mühlebach et al. attribute this to increased crosslink density.^[^ ^6^ ^]^ The pyrolytic degradation properties in relation to the aging of the samples are not discussed in the respective publications. Steese et al. observes reduced volatilization during pyrolysis of PDCPD samples with a higher cross–linking.^[^ ^10^ ^]^ It is thus plausible that the pyrolytic degradation properties change with the sample aging history. However, the aging procedure performed in this study appears to have little influence on the degradation behavior in TGA. Assuming higher crosslink density in the aged sample, one would expect an increased tendency to form char. However, PDCPDa forms consistently less solid residue. Dimonie et al. have detected thermal polymerization of macromolecular double bonds at 150 to 200 °C.^[^ ^11^, ^12^ ^]^ Similarly, Vidavsky et al report secondary metathesis reactions of olefins above 150 °C with a parallel increase of the glass transition temperature.^[^ ^9^ ^]^ With a lower heating rate, the TGA sample experiences increased temperatures for a longer time before the onset of volatile release. Hence, it is plausible that at lower heating rates, the polymer undergoes more pronounced polymerization and cross–linking. In turn, this may promote residue formation as observed in the presented analyses. Furthermore, one may assume that the effect of the aging procedure of PDCPDa is mitigated during the heat‐up phase of the samples in TGA with the occurrence of thermal polymerization. Multiple studies have shown that PDCPD is prone to oxidation, especially when exposed to thermal stress.^[^ ^8^, ^13^, ^14^ ^]^ The increased oxidation expected for PDCPDa is a plausible cause for the lowered solid yield. The previously oxidized polymer may form CO_2_ during pyrolysis, which lowers the solid yield. The mechanisms behind these observed phenomena cannot be resolved from TGA alone. For a more detailed investigation of the volatile products formed in TGA, hyphenated techniques such as TG‐FTIR should be employed. Tracing methods for characteristic bands allow time‐resolved observation and quantification of certain products such as carbon dioxide.^[^ ^15^ ^]^

### 2.2 Py‐GC‐MS

**Figure 2:**
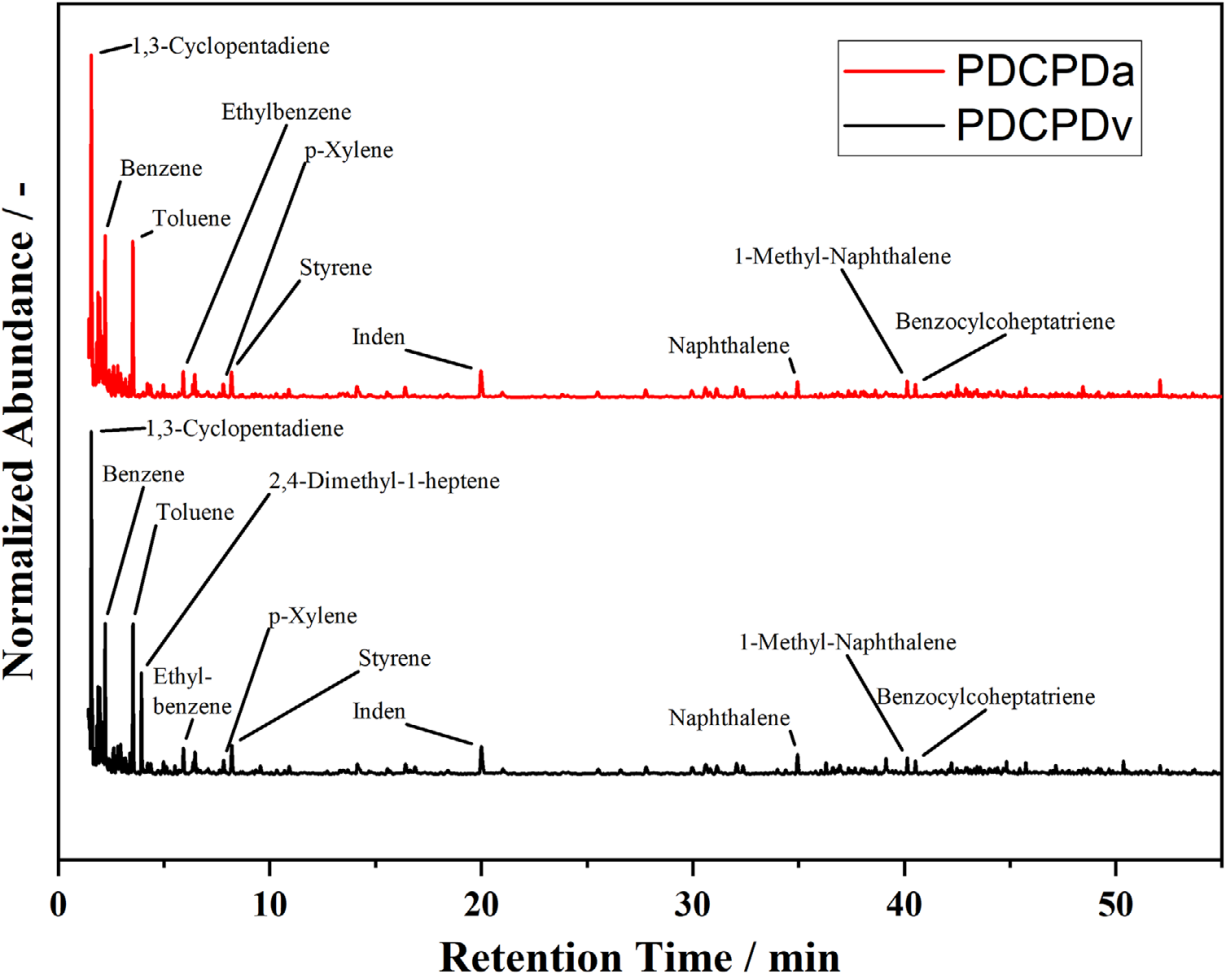
Chromatogram of PDCPDv and PDCPDa pyrolysis from Py‐GC‐MS experiments at 800 °C and a pyrolysis time of 1 min. Chromatogram cut after 55 min, full analysis runtime is 74 min.

**Figure** **2** shows the pyrograms of non‐pretreated PDCPDv and artificially aged PDCPDa. The product spectra and relative abundances are virtually identical. No significant influence of the aging procedure is detectable. This agrees with the general findings from TGA.

It is evident from the pyrograms that the product spectrum is broad and contains predominantly cyclic and aromatic hydrocarbons. 1,3‐Cyclopentadiene (1,3‐CPD) is detected as the substance with the highest abundance. The 1,3‐CPD peak is followed by multiple smaller peaks belonging to cyclic hydrocarbons with carbon numbers of 5 and 6. Benzene, Toluene, Ethylbenzene, and p‐Xylene are also prominent products. Inden, naphthalene, and naphthalene derivates are also detected. Mühlebach et al. have found 1,3‐CPD to be the dominant pyrolysis product besides monoaromatics, indenes and indanes and naphthalenes.^[^ ^6^ ^]^ This agrees well with the findings presented in this publication.

The chemical structure of the PDCPD contains cyclic hydrocarbon segments and is unsaturated. Chemical bonds that cleave during pyrolysis cannot be saturated due to the hydrogen deficiency in the polymer. Thus, the formation of double bonds, cyclic hydrocarbons, and aromatics is conclusive. The predominant pyrolytic decomposition mechanism of PDCPD is the depolymerization to 1,3‐CPD. DCPD was not detected. DCPD is known to disintegrate to 1,3‐CPD at temperatures above 170 °C. Conversely, 1,3‐CPD dimerizes to DCPD at room temperature.^[^ ^16^ ^]^ Since the Py‐GC‐MS system, the transfer line to the GC, and the GC itself are heated above 170 °C, the formation of DCPD appears to be suppressed. A quantitative assessment of the product spectrum and distribution cannot be given by Py‐GC‐MS. In the quartz glass tubes that carry the sample, a visible residue remains. This indicates that the sample cannot be completely volatilized. For Py‐GC‐MS it can be assumed that heat and mass transfer limitations are low. After volatilization, the pyrolysis products are immediately cooled and heavily diluted by the He carrier gas flow. It can be assumed that this suppresses secondary pyrolysis reactions. Nonetheless, the formation of a residue is evident. Thus, significant solids formation appears to be an intrinsic property of the PDCPD in pyrolysis.

### 2.3 Dynamic Lab‐Scale Batch Experiment

**Figure 3:**
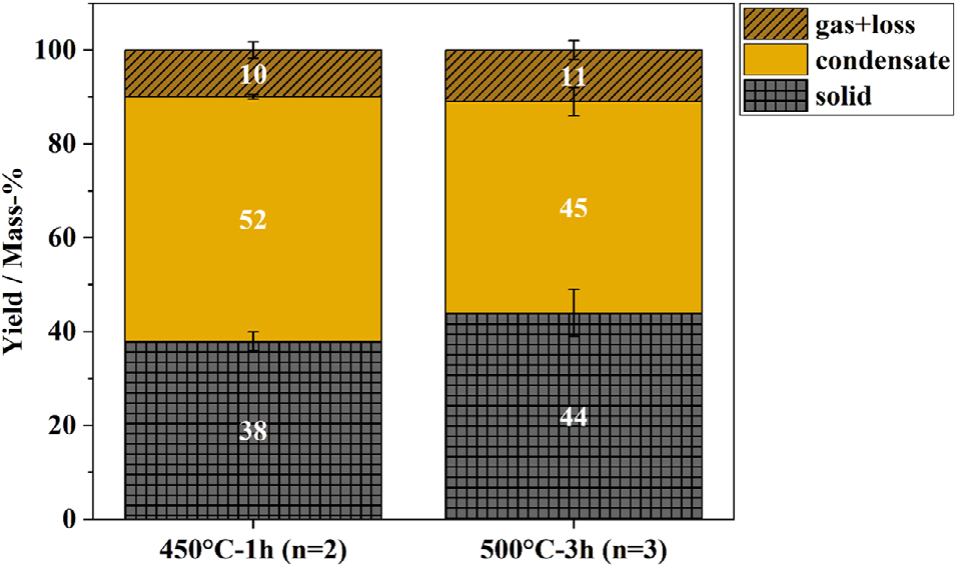
Mass balances of lab‐scale batch experiments of PDCPDv at different temperatures and isothermal dwell times. Error bars represent the standard error.

Experiments with isothermal temperatures of 450 and 500 °C were conducted. For the experiments at 450 °C, an isothermal dwell period of 1 h was set. The 500 °C experiments were conducted with an isothermal dwell period of 3 h. The TGA and Py‐GC‐MS experiments show marginal differences between PDCPDv and PDCPDa. Therefore, only PDCPDv was further investigated in the lab‐scale setup. **Figure** **3**. provides an overview of the conducted experiments and the corresponding mass balances.

**Figure 4:**
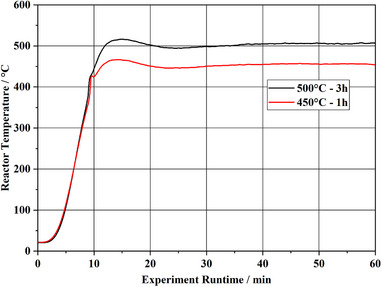
Reactor Temperature profiles of experiments with PDCPDv at 450 and 500 °C during the heat‐up phase of the system.

For the experiment 450 °C‐1 h, the system was heated to a temperature of 450 °C and held for 1 h after the temperature setpoint was reached. 38% of the original sample mass was converted to solids. For 500 °C‐3 h, 44% solids are produced. The obtained solids from all experiments consist of char and a sticky, tarry black residue. Between 45 and 52 mass‐% of the sample is converted to condensate. With 10 to 11 mass‐%, the yield of gas is small. Due to the high solid yield from the experiments at 450 °C, it was concluded that the isothermal temperature is too low to achieve satisfactory conversion in a reasonable pyrolysis time. Therefore, the isothermal temperature and the dwell time were increased. For the 500 °C experiment with a 3 h dwell time, the solid yield was higher than in the experiments at 450 °C with a dwell time of 1 h. The hypothesis of an incomplete pyrolysis reaction at 1 h dwell time may thus be considered invalid. The general solid formation appears to be only minorly dependent on the pyrolysis temperature. From TGA it is evident that volatilization starts at ca. 100 °C. The onset of the main volatilization step is around 350 to 400 °C. This means that the pyrolysis reactions in the lab scale system must already commence during the heating phase of the system. Due to the dynamic nature of the experiment, each sample is subject to a heating phase. The recorded reactor temperatures during the heating phase are given in **Figure** **4**.

In the heating phase, each sample experiences the same thermal energy input, regardless of the final temperature. Higher pyrolysis temperatures promote thermal cracking reactions, which are expected to lead to a higher fraction of gas and volatiles.^[^ ^17^ ^]^ As Figure 3 shows, this is not of effect in the presented experiments. The distribution of solids, condensables, and non‐condensables changes only slightly. Nonetheless, it is possible that a shift from char to tar‐like residue occurs with variation in the pyrolysis temperature. With the employed system and experimental method, no quantitative distinction between tar and char can be made. The formation of condensable pyrolysis products and permanent gases is low compared to other common pure and mixed plastic wastes.^[^ ^17^, ^18^, ^19^, ^20^ ^]^

A significant deviation from the TGA findings is evident. While only ca. 20 mass‐% of the polymer is converted to solids in TGA, the solid yield on lab‐scale ranges from 38 to 44 mass‐%. This increase in solids corresponds to a reduced yield of condensates and permanent gases. A possible reason for this difference is the influence of heat and mass transfer and secondary pyrolysis reactions. The TGA oven containing the crucible with the sample is constantly flushed with 60 mL min^−1^ of N_2_. Evolving volatiles are immediately diluted and carried out of the oven. It can be assumed that secondary reactions in the gas phase are suppressed by this. In contrast, the lab‐scale reactor is constantly flushed with 50 mL min^−1^ of inert gas. Additionally, while the initial sample mass in TGA is 10 µg, it amounts to several grams in the lab‐scale system. As a result, the dilution effect on lab‐scale is weaker, and evolving volatiles remain in the reactor's hot zone for a longer time compared to TGA. This increases the likelihood of secondary and recombination reactions, which may explain the increased tendency to form tar and char.^[^ ^17^ ^]^

**Figure 5:**
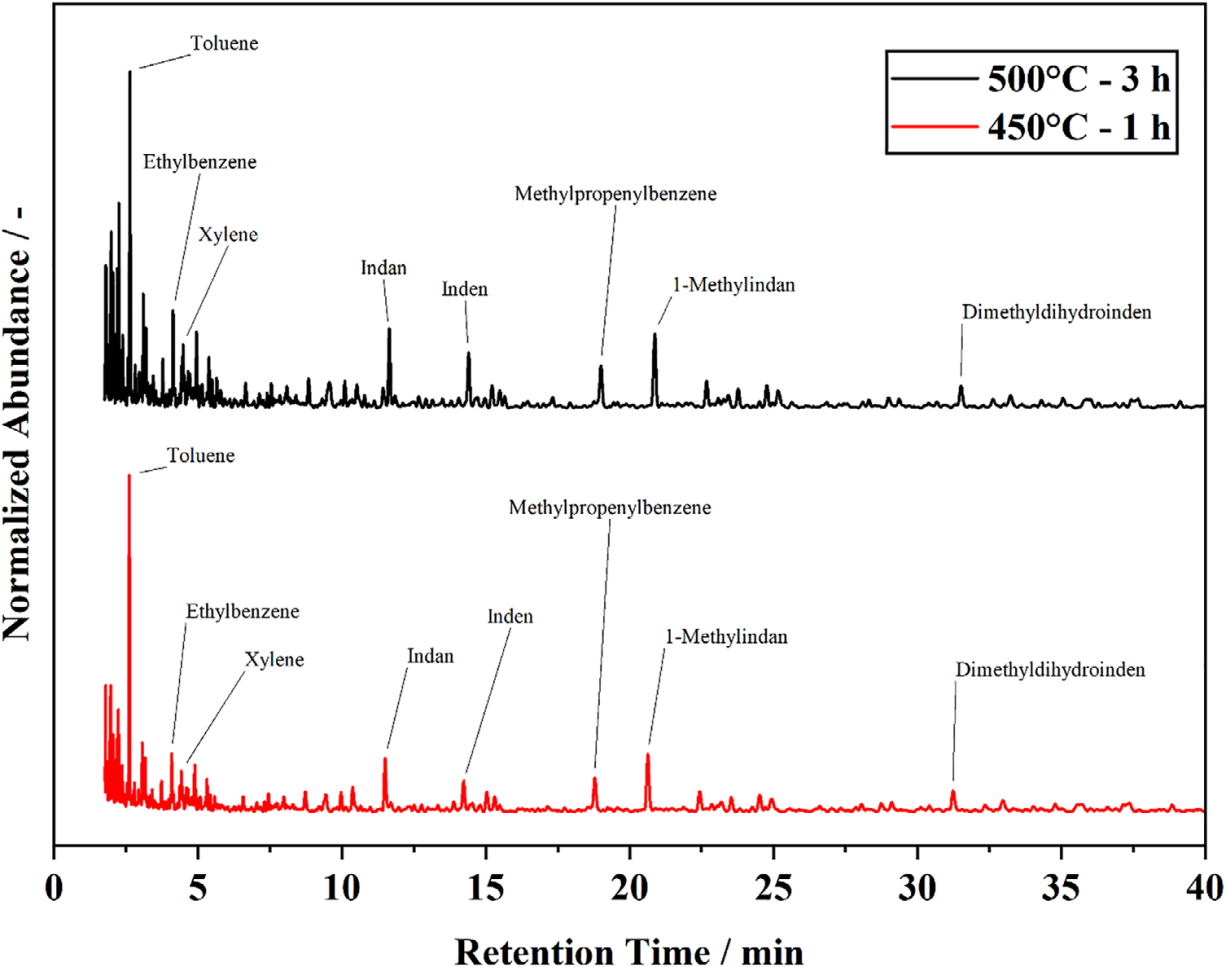
GC‐MS‐Chromatogram of PDCPDv condensates obtained from lab scale pyrolysis at 500 °C and 3 h isothermal dwell time and 450 °C and 1 h isothermal dwell time. Chromatogram cut after 40 min, full analysis runtime 265 min.

With the employed system, adequate condensate quantities for offline analyses can be collected. The chromatographic analysis of the condensate obtained from pyrolysis at 500 °C and 3 h isothermal dwell time is shown in **Figure** **5**.

The GC‐MS analysis of the condensate reveals a broad product spectrum. The chromatograms of the condensates obtained from the two different experimental setpoints are nearly identical. Thus, the variation of the pyrolysis temperature and the dwell time has little influence on the product spectrum. Indan, inden, and their derivates stand out. Also, octane, toluene, and ethylbenzene show high abundance. In the retention time region below 5 min, cyclic alkanes and alkenes are detected in high abundance. The library search results for most peaks in this retention time region are ambiguous and not certainly attributable to a specific substance. For these peaks, cyclic alkanes and alkenes achieve very similar match factors. The products of an eventual depolymerization, i.e., 1,3‐CPD and DCPD, are not detected. In this regard, the product spectrum at lab‐scale deviates from the products obtained with the Py‐GC‐MS. From the lab‐scale mass balance and the GC‐MS analysis, it appears that secondary reactions dominate the observed pyrolytic behavior. If formed, the depolymerization products 1,3‐CPD and DCPD undergo secondary reactions to mainly produce tar and char. This is in line with the obtained mass balances and the deviation from TGA. A fraction of the pyrolysis products volatilize and are removed from the reactor. Whether the substances in the condensate are subject to further derivatization or repolymerization cannot be concluded with the employed system. Considering the amount of unsaturated and cyclic compounds in the condensate, recombination reactions are plausible. In the Py‐GC‐MS system, the evolved pyrolysis products are immediately removed from the pyrolysis chamber and heavily diluted by He. Thus, secondary reactions are suppressed, which explains the differences in the observed product spectra at µg‐ and lab‐scales.

At the lab and technical scale, pyrolysis initially generates a solid residue fraction and a vapor product fraction. Typically, the vapor fraction is cooled in a downstream condensation unit and separated into a condensed and a non‐condensed product fraction. Species with boiling points above the condenser temperature mostly condense and consequently, make up the condensate. Due to vapor‐liquid equilibrium effects, fractions of these compounds are also found among the non‐condensable products, together with the permanent gases produced from pyrolysis. This cannot be avoided entirely. Thus, it is necessary to employ robust analytical equipment that is capable of analyzing both permanent gases and longer‐chained hydrocarbons in the non‐condensed product phase. Commonly used systems for gas analysis, such as µGCs, are suitable for permanent gases and short‐chain hydrocarbons below C_5_. Therefore, there is a risk that the product range of non‐condensables cannot be fully covered. Furthermore, they lack an MS detector and its identification capabilities. For commonly broad pyrolysis product spectra, thorough identification is essential.

**Figure 6:**
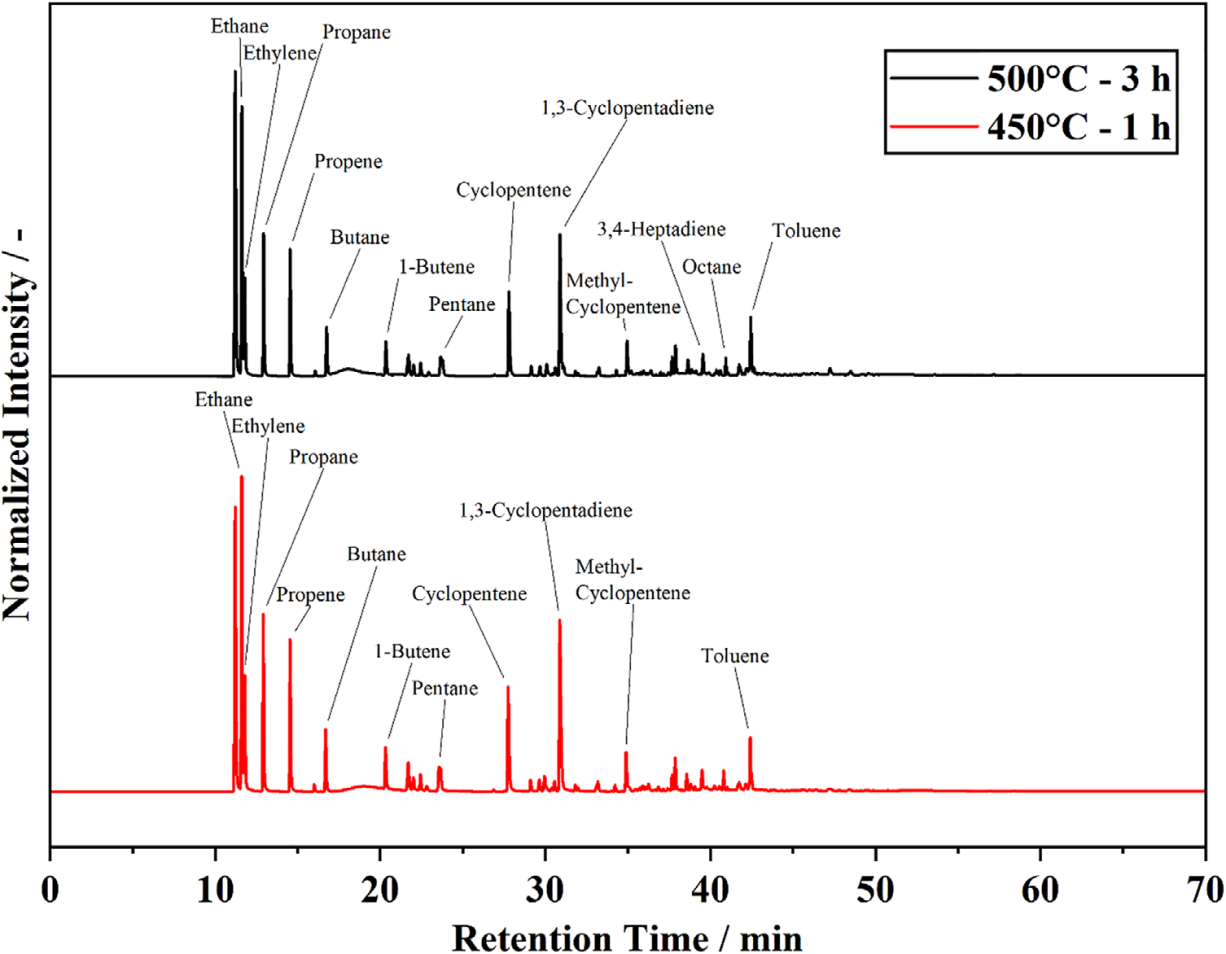
GC‐FID‐Chromatograms of PDCPDv non‐condensables obtained from lab‐scale pyrolysis at 500 °C and 3 h isothermal dwell time and 450 °C and 1 h isothermal dwell time. Identification was performed with parallel GC‐MS.

The non‐condensable product fraction from lab‐scale pyrolysis was collected in gas bags and analyzed by GC‐FID‐TCD‐MS. With the employed equipment, a comprehensive analysis of the short‐chain hydrocarbon product spectrum is possible. The resulting chromatogram from the GC‐FID‐MS strain is shown in **Figure** **6**.

The analysis presented in Figure 6 shows the expected overlap between the condensate and the non‐condensables analysis. Short‐chain saturated and unsaturated hydrocarbons from C_1_ to C_8_ are detected. Substances like toluene and ethylbenzene, which are present in the non‐condensable product fraction, are also detected in high abundance in the condensate. Substances of particularly high abundance are cyclopentene, 1,3‐CPD, and methyl‐cyclopentene. As already determined by the condensate analysis, the variation of the pyrolysis temperature and the dwell time has no influence on the product spectrum. The displayed chromatograms of the non‐condensables analysis are qualitatively identical. The analysis is generally consistent with Py‐GC‐MS and the condensate analysis. Pyrolysis of PDCPD produces mainly cyclic and unsaturated hydrocarbons. Contrary to the condensate analysis, 1,3‐CPD is detected in the gas phase as a product of PDCPD pyrolysis. This hints at secondary reactions during or after condensation. It is plausible that the pyrolytically formed 1,3‐CPD undergoes further reactions and is thus not found in the condensate analysis. Since no DCPD is found in the condensate, it can be assumed that 1,3‐CPD dimerization is minimal, or that DCPD undergoes derivatization reactions. The TCD strain of the analyzer shows distinct peaks for carbon dioxide and methane. The detected carbon dioxide may be formed from O adsorbed to the polymer surface or from O‐containing additives. When kept under an air atmosphere, PDCPD is prone to oxidation.^[^ ^13^, ^21^ ^]^ The investigated sample was stored at room temperature in air. Consequently, it is expected that the investigated sample has experienced oxidation. Therefore, the formation of CO_2_ in pyrolysis is conclusive, despite the inert pyrolysis atmosphere.

### 2.4 Implications for PDCPD Recycling

In the context of chemical recycling of polymers, the recovery of monomers, leading to closed‐loop recycling, is often the desired pathway.^[^ ^22^ ^]^ It has been proven that pyrolysis is capable of high‐yield monomer recovery with certain polymers such as polystyrene and polymethylene methacrylate.^[^ ^18^, ^20^, ^23^ ^]^ While Mühlebach et al. and the Py‐GC‐MS findings in the presented study indicate monomer generation from PDCPD as a predominant mechanism, monomer recovery was not achieved in lab‐scale pyrolysis.^[^ ^6^ ^]^ The temperature and pyrolysis duration were varied without significant influence on the yield and the product spectrum. One can expect that further variation of the pyrolysis conditions will not achieve a significant shift toward high‐value condensed and gaseous products. Several options for chemical recycling of PDCPD via pyrolysis remain. Gasification can serve as a purposeful outlet for pyrolysis products. Hennig et al. have demonstrated the potential of combined pyrolysis and gasification for complex mixed plastic waste.^[^ ^24^ ^]^ The advantages of gasification are the potential use of solid and condensed pyrolysis product fractions and the high heteroatom tolerance. Combined gasification of solid and condensed biomass pyrolysis products was demonstrated on a demo scale in an entrained flow gasifier.^[^ ^25^ ^]^ The use of PDCPD pyrolysis solids and condensates in such a gasification system is plausible but has to be investigated further. While no direct monomer recovery is possible, carbon recovery rates are expected to be higher than with standalone pyrolysis. In that case, the pyrolysis solids must be considered lost from a circularity perspective. Another potential option to optimize the yield of PDCPD pyrolysis is the employment of a catalyst. Catalytic pyrolysis is widely studied with promising results for various plastic types and waste streams. To the best of our knowledge, no studies on catalytic PDCPD pyrolysis have been presented yet. The strengths of a catalyst employed for such a task should comprise the suppression of solids formation, shifting toward condensate production, and derivatization of the condensate compounds to stable, valuable compounds, less prone to recombination. Multiple recent studies have elaborated on the application of catalysts for the pyrolysis of polyolefins and mixed plastic wastes.^[^ ^26^, ^27^, ^28^ ^]^ Furthermore, catalysts that are employed in the refinery are an option.^[^ ^29^ ^]^ However, relevant catalysts have to be assessed regarding their compatibility with PDCPD feedstock and pyrolysis conditions.

In the sense of a circular economy, the utilization of pyrolysis condensates in steam cracking is also an option. Fossil naphtha is typically used as a feedstock, which is cracked to short‐chain olefins in the process. Naphtha can potentially be replaced by pyrolysis condensates. However, steam cracker feedstock specifications must be strictly adhered to, which is challenging for many plastic waste pyrolysis condensates.^[^ ^5^ ^]^ The expected low heteroatom contamination of PDCPD pyrolysis condensate renders it a potential feedstock for steam cracking. However, further research is to be conducted to evaluate combined pyrolysis and steam cracking recycling routes.

## 3 Conclusion

In the presented work, we investigated the pyrolysis of PDCPD by Py‐GC‐MS, TGA, and dynamic lab‐scale batch pyrolysis. Differences in the decomposition characteristics on each scale are evident. The polymer exhibits a pronounced tendency to form a solid or tarry residue. 1,3‐CPD was detected in Py‐GC‐MS and the non‐condensables analysis at lab‐scale, which hints at depolymerization as the initial decomposition mechanism. The absence of 1,3‐CPD and its dimer DCPD in the obtained condensate proves the influence of secondary reactions in the reaction system and the subsequent units, such as the condenser. Furthermore, an influence on the product yields and compound spectra in the respective phases is evident. The significantly increased tendency to form solid residue at lab‐scale underlines this. To establish pyrolysis as a recycling approach, effective ways to shift the product yields toward condensate and gas, propagate the depolymerization, and suppress secondary reactions are to be found. The aging of PDCDP was found to be insignificant to the general decomposition characteristics, aside from a slight influence on the tendency to form solid residue, as observed in TGA. With the presented experimental and analytical approach, we underline the importance of an investigation of pyrolysis characteristics across multiple scales and the support of high‐resolution analytics. Independent of the investigated polymer, this enables the initial assessment of primary and secondary decomposition pathways and product formation mechanisms. Possible system artifacts can be detected, and scaling phenomena discussed. The generation of a profound understanding of decomposition products, product yields, and crucial process parameters is essential for the critical evaluation of pyrolysis as a recycling option. The presented approach and analytical toolset serve as a starting point for the development of robust commercial‐scale processes.

## 4 Experimental Section

### Sample Preparation

Polydicyclopentadiene sheets were provided by Metton America Inc. Two samples were prepared by mixing liquid molding resin in an impingement mixer and subsequent reaction injection molding. The sample PDCPDv was used without further treatment, while the PDCPDa sample was artificially aged in an oven at 90 °C for 14 days. The PDCPD sheet samples were hand‐cut and further rotor milled to a particle size below 500 µm using a Fritsch Pulverisette 14.

### Thermogravimetry

For the thermogravimetric analyses, a Netzsch TG 209 F1 Libra with an automatic sample changer was employed. Per the experiment, ≈10 mg of the sample was analyzed in corundum crucibles without a lid. Dynamic experiments from 30 to 900 °C with heating rates of 10, 20, and 40 K min^−1^ were conducted. The system was constantly flushed with N_2_ at a flow rate of 60 mL min^−1^. After the termination of the pyrolysis experiment at 900 °C, the atmosphere was switched to synthetic air for oxidation of residues and deposits in the oven chamber. Each experiment was carried out three times.

### Py‐GC‐MS

For Py‐GC‐MS experiments, a CDS Pyroprobe 6200 DISC micropyrolyzer coupled to an Agilent 7890B Gas Chromatograph (GC) and an Agilent 5977B Mass spectrometer (MS) was used. Per the experiment, between 100 and 200 µg of sample was pyrolyzed. The pyrolysis was operated at temperatures of 600 °C or 800 °C for 60 s under He flow. The pyrolysis products were swept to the GC through a transfer line heated to 350 °C to minimize condensation. Separation in the GC was performed by a Restek RTX200MS column (31 m, 0.25 mm ID 0.25 µm film thickness). The GC inlet was set to the maximum temperature of 300 °C in split mode with a split of 100:1, and a constant pressure of 70 kPa was applied. The oven temperature was held at 35 °C for 5 min and then ramped to 60 °C at a rate of 1 °C per min. Subsequently, the oven temperature was ramped to 280 °C at 5 °C per minute, resulting in a total runtime of the analysis of 74 min.

### Dynamic lab‐scale batch experiment

**Figure 7:**
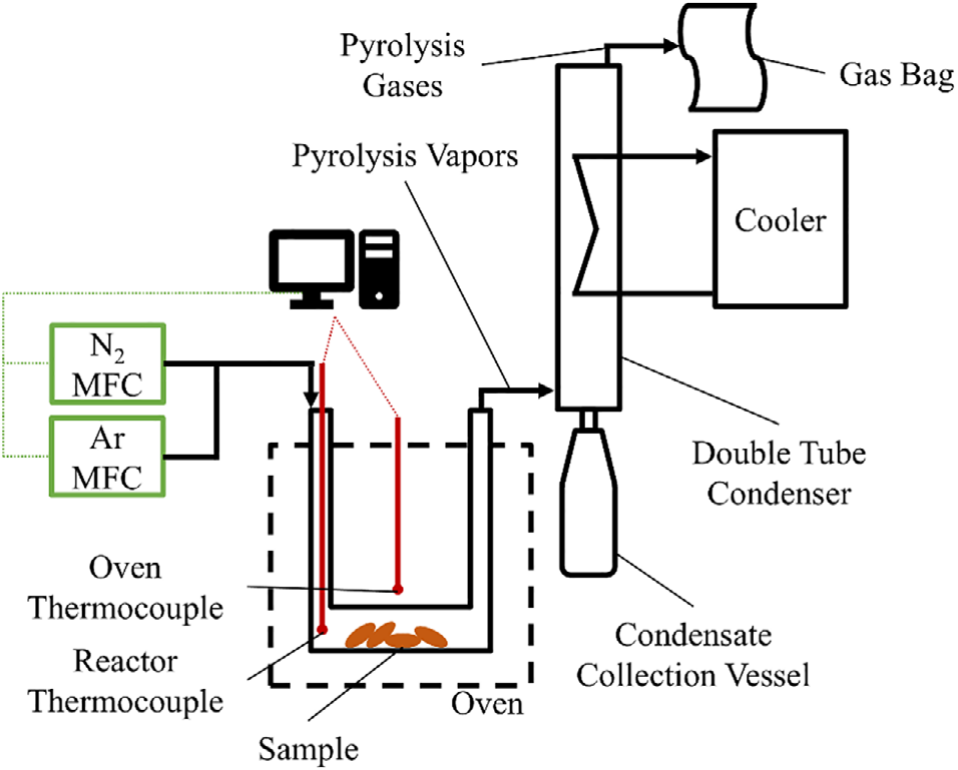
Schematic Drawing of the lab‐scale batch pyrolysis system.

For experiments on lab‐scale, a batch pyrolysis reactor system was used. The setup is displayed in **Figure** **7**.

The pyrolysis system consists of mass flow controllers for N_2_ and Ar dosing, a U‐shaped reactor placed in an electrically heated oven, a double tube condensation unit, and a gas collection unit. The oven and the reactor thermocouple record the temperature of the oven and the reactor interior, respectively. The oven was connected to the reactor thermocouple and was controlled to maintain a constant internal reactor temperature. Prior to each experiment, the reactor, the connection pipes, and the condensation unit were weighed. Between 3.0 and 4.5 g of sample was then filled into the reactor. The reactor and condensation unit were sealed airtight and flushed with inert gas for several minutes. The cooler of the condensation unit was set to ‐5 °C, employing a mix of water and ethylene glycol as a coolant. The oven was then heated to the desired pyrolysis temperature. After reaching the set temperature, the pyrolysis was conducted for a defined period of time. The evolved products of the pyrolysis were constantly flushed to the condensation unit by a flushing gas flow of 50 mL min^−1^. The flushing gas consists of N_2_ and Ar in a defined ratio. Non‐condensable products of the pyrolysis were collected in a RESTEK Tedlar gas bag for analysis. After the end of the experiment, the oven was switched off and the reactor was removed to quench the pyrolysis. The system was disassembled and weighed again. To ensure accurate weighing, the inner tube of the condenser can be removed. Thus, weighing errors from coolant deposits in the condenser were avoided. From the weight difference, the mass balance was derived. Products found in the connector, the condenser, and the connection tube to the gas bag were considered condensates, while the material remaining in the reactor was registered as solid residue. The mass of the produced gas was calculated by difference. Thus, it comprises all potential losses and uncertainties. After weighing, the condensation unit was disassembled, and the condensate was collected in vials. The system was then washed with Chloroform (CHCl_3_). Insoluble deposits in the reactor were burned off by heating the reactor with a gas burner in the air atmosphere.

### Condensate Analysis by GC‐MS

An Agilent 6890 GC coupled to a 5973 Quadrupole Mass Spectrometer with a Restek RTX200MS column (30 m, 0.25 mmID, 0.25 µm film thickness) was employed for the analysis of pyrolysis condensate. Sample preparation for the analysis was performed by dilution with CHCl_3_ in a ratio of 1:5. 1 µL: of the diluted sample was injected into the GC by an automatic liquid sampler. The split/splitless‐inlet was heated to 270 °C and flushed with He at a pressure of 75 kPa. The split ratio was set to 60:1 with a total flow of 87 mL min^−1^. During the analysis, the oven temperature was initially held constant at 40 °C for 5 min. Subsequently, the oven temperature was ramped at 1 °C min^−1^ to the final temperature of 300 °C. The total runtime of the analysis was 265 min. The MS was operated in scan mode. The MS transfer line and the ion source were heated to 250 and 230 °C, respectively. The quadrupole temperature was 150 °C.

The chromatograms were manipulated using Lablicate OpenChrom. For the MS spectrum library search, the NIST 17 spectral database was employed. Only peaks with > 80% match factor were considered as identifiable.

### Non‐condensables Analysis by GC‐FID‐TCD‐MS

**Figure 8:**
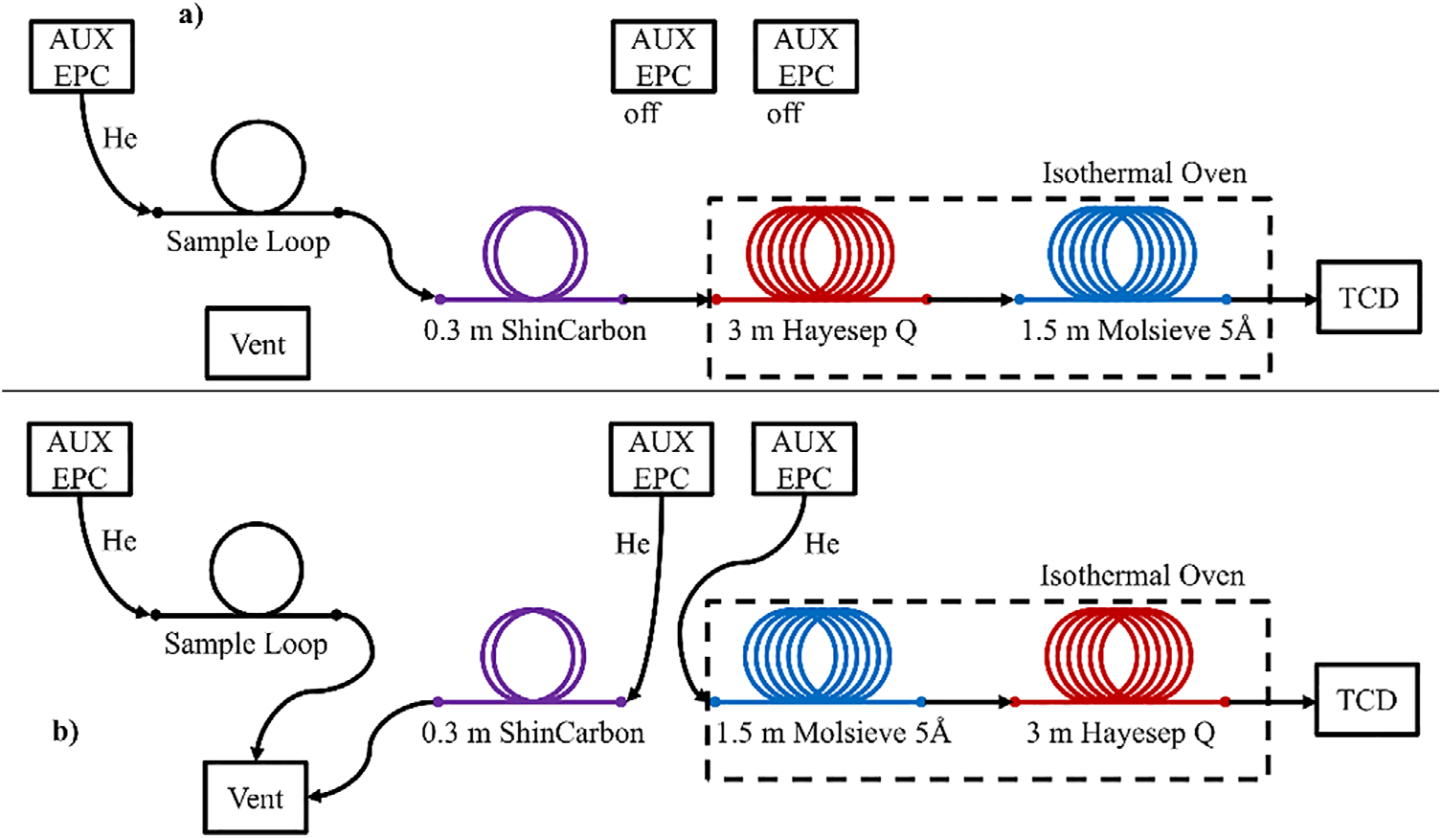
Gas Flow Pathways for TCD analysis. a) initial column configuration, b) ShinCarbon backflush and reversed column configuration.

An Agilent 8890 GC with a customized configuration was employed for the analysis of non‐condensable pyrolysis products. The GC system was customized by Teckso GmbH. It was equipped with an automatic gas sampling unit, two sample loops, multiple switching valves, an additional isothermal column oven, and multiple columns connected via dean switches. A thermal conductivity detector (TCD), a flame ionization detector (FID), and an Agilent 5977C mass spectrometer S were employed. This setup enables high separation efficiencies and the detection and identification of crucial products. The system was fed with gas samples from Restek Tedlar bags. Initially, the sample loops were filled with the sample via a suction nozzle. One sample loop and the column sequence ShinCarbon, Hayesep Q, and Molsieve were attached to the TCD. The Hayesep Q and the Molsieve column were situated in an isothermal oven, which was constantly held at 50 °C. The initial configuration is shown in **Figure** **8**a). With the start of the analysis run, the sample was flushed onto the ShinCarbon column where compounds with higher boiling points were retained. Ar, O_2_, N_2_, CO, CO_2,_ and CH_4_ pass through and were separated in the Hayesep Q and Molsieve columns. H_2_, Ar, and N_2_ first eluted from both columns and were detected within the first 5 min of the GC run. CO_2_, CH_4,_ and CO remain on the Molsieve column. The flow and column configuration were then switched as displayed in Figure 8b). This achieves a backflush of the ShinCarbon column to the vent and thus protects the subsequent columns against contamination. The order of the Hayesep Q and Molsieve columns was reversed. The gases retained on the Molsieve column elute, pass through the Hayesep Q column again, and were detected in the TCD after the final elution. This increases the separation efficiency and enables a more accurate analysis. The analysis on this strain was completed after the elution of CO for 14 min. The system remains in state b) until the end of the analysis run.

**Figure 9:**
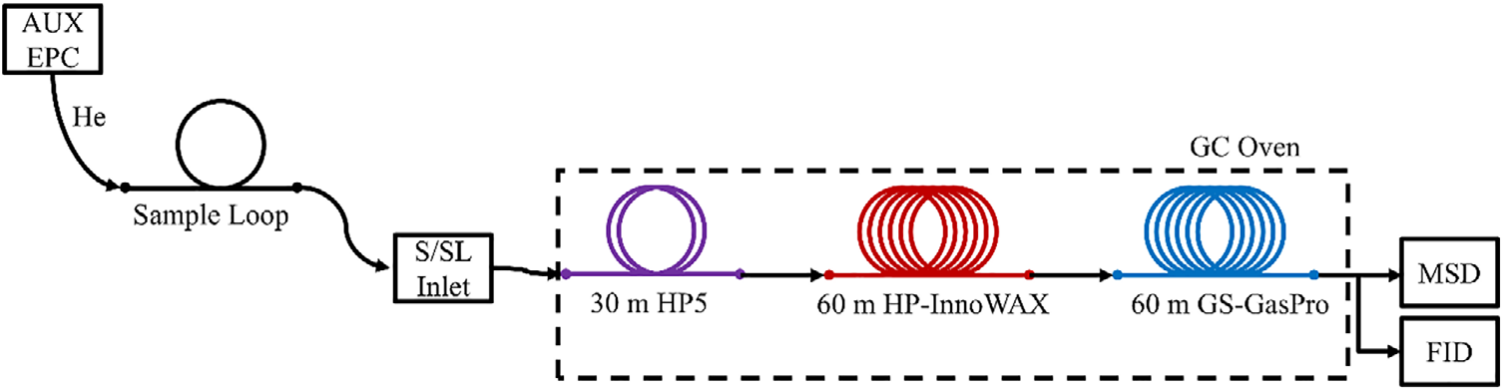
Column Configuration for FID and MS gas analysis.

For the separation of higher molecular weight hydrocarbon gases, the second sample loop attached to a cascade of an HP5, an Innowax, and a GasPro column was employed. The setup and gas flow pathway are shown in **Figure** **9**. The HP5 and Innowax columns provide separation according to the boiling point/chain length of the analytes. The eluting gas stream was split and fed to the MS and the FID. This enables identification via mass spectra database comparison and reliable quantitation via the FID.

The oven program is given in **Table** **1**. A temperature profile with multiple ramps was used to achieve optimal separation. The temperature of the split/splitless inlet was 250 °C. The inlet pressure was 2.5 bar with a flow setpoint of 35 mL min^−1^ and a split ratio of 5:1. The system was operated in constant pressure mode. The overall runtime of 70 min of the analysis method was determined by the runtime of the FID and MS analysis strain.

**Table 1:** Oven Temperature Program.

Heating	End	Hold	Run
rate °C min^−1^	temperature °C	time min	time min
–	45	1	1
3	114	0	24
5	174	0	36
10	220	29.4	70

## Conflict of Interest

The authors declare no conflict of interest.

## Data Availability

The data that support the findings of this study are available from the corresponding author upon reasonable request.

## References

[1] M. Bachmann , C. Zibunas , J. Hartmann , V. Tulus , S. Suh , G. Guillén‐Gosálbez , A. Bardow , Nat. Sustain. 2023, 6, 599.

[2] J. C. Cooper , J. E. Paul , N. Ramlawi , C. Saengow , A. Sharma , B. A. Suslick , R. H. Ewoldt , N. R. Sottos , J. S. Moore , Adv. Mater. 2024, 36, 2402627. 10.1002/adma.202402627 38652482

[3] Z. Xu , K. Wang , B. A. Suslick , J. S. Moore , J. Am. Chem. Soc. 2025, 147, 8732. 39999420 10.1021/jacs.4c18018

[4] K. Liu , M. E. Battson , Z. Hu , Y. Zhao , E. M. Rettner , J. Miscall , N. A. Rorrer , G. M. Miyake , Angew Chem Int Ed Engl. 2025, 64, 202423111. 10.1002/anie.202423111 39824761

[5] M. Kusenberg , A. Eschenbacher , M. R. Djokic , A. Zayoud , K. Ragaert , S. de Meester , K. M. van Geem , Waste Manage. 2022, 138, 83. 10.1016/j.wasman.2021.11.009 PMC8769047 34871884

[6] A. Mühlebach , P. van der Schaaf , A. Hafner , F. Setiabudi , J. Mol. Catal. A: Chem. 1998, 132, 181.

[7] E. Richaud , P. Y. Le Gac , J. Verdu , Polym. Degrad. Stab. 2014, 102, 95.

[8] J. Huang , W. Minne , R. Drozdzak , G. Recher , P. Y. Le Gac , E. Richaud , Polym. Degrad. Stab. 2020, 174, 109102.

[9] Y. Vidavsky , Y. Navon , Y. Ginzburg , M. Gottlieb , N. G. Lemcoff , Beilstein J. Org. Chem. 2015, 11, 1469. 26425203 10.3762/bjoc.11.159 PMC4578353

[10] N. D. Steese , D. Barvaliya , X. D. Poole , D. E. McLemore , J. C. DiCesare , H.‐J. Schanz , J. Polym. Sci. Part A: Polym. Chem. 2018, 56, 359.

[11] D. Dimonie , M. Dimonie , V. Munteanu , H. Iovu , J. Couve , M. J. Abadie , Polym. Degrad. Stab. 2000, 70, 319.

[12] D. Dimonie , M. Dimonie , S. Stoica , V. Munteanu , M. J. Abadie , Polym. Degrad. Stab. 2000, 67, 167.

[13] V. Defauchy , P. Y. Le Gac , A. Guinault , J. Verdu , G. Recher , R. Drozdzak , E. Richaud , Polym. Degrad. Stab. 2017, 142, 169.

[14] J. Huang , A. David , P.‐Y. L. Gac , C. Lorthioir , C. Coelho , E. Richaud , Polym. Degrad. Stab. 2019, 166, 258.

[15] M. Zeller , D. Merz , L. Weigel , S. Tavakkol , D. Stapf , J. Anal. Appl. Pyrolysis 2025, 188, 107048.

[16] P. J. Wilson , J. H. Wells , Chem. Rev. 1944, 34, 1.

[17] H. C. Genuino , M. C. P. van Eijk , S. R. A. Kersten , M. P. Ruiz , Energy Fuels 2024, 38, 2188.

[18] N. Netsch , M. Simons , A. Feil , H. Leibold , F. Richter , J. Slama , S. P. Yogish , K. Greiff , D. Stapf , Waste Manage. 2022, 150, 141. 10.1016/j.wasman.2022.07.001 35834862

[19] J. Vogt , A. Renno , M. C. Fuchs , T. Kurtz , N. Netsch , F. Richter , G. Straczewski , B. Bergfeldt , Y. Madriz‐Diaz , A. de Lima Ribeiro , D. Ebert , S. Tavakkol , S. Raatz , D. Stapf , Mechanical Engin. 2024.

[20] M. Zeller , N. Netsch , F. Richter , H. Leibold , D. Stapf , Chemie Ingenieur Technik. 2021, 93, 1763.

[21] A. A. Lyapkov , E. L. Gvozdkov , A. N. Tarakanovskaya , O. D. Tarnovskaya , Y. S. Yakovleva , Procedia Chem. 2014, 10, 223.

[22] G. W. Coates , Y. D. Y. L. Getzler , Nat. Rev. Mater. 2020, 5, 501.

[23] W. Kaminsky , J. Franck , J. Anal. Appl. Pyrolysis 1991, 19, 311.

[24] M. Hennig , T. Dreising , A. Reeves , T. Oehlcke , S. Tavakkol , R. Volk , F. Schultmann , D. Stapf , ACS Sustainable Chem. Eng. 2025.

[25] M. Eberhard , U. Santo , B. Michelfelder , A. Günther , P. Weigand , J. Matthes , P. Waibel , V. Hagenmeyer , T. Kolb , ChemBioEng Rev. 2020, 7, 106.

[26] M. Seitz , S. Schröter , Chem. Ing. Tech. 2022, 94, 720.

[27] N. Netsch , J. Vogt , F. Richter , G. Straczewski , G. Mannebach , V. Fraaije , S. Tavakkol , S. Mihan , D. Stapf , Chem. Ing. Tech. 2023, 95, 1305.

[28] K. Akubo , M. A. Nahil , P. T. Williams , J. Energy Inst. 2019, 92, 195.

[29] A. Galadima , O. Muraza , Fuel 2016, 181, 618.

